# First human case of *Eidernor doerrieniae* colonization in a peritoneal dialysis catheter: A warning from silent contamination

**DOI:** 10.1016/j.mmcr.2025.100696

**Published:** 2025-02-11

**Authors:** Phichit Songviriyavithaya, Aschariya Wipattanakitcharoen, Niparat Pikul, Dhammika Leshan Wannigama, Talerngsak Kanjanabuch

**Affiliations:** a Division of Nephrology, Department of Medicine of Amnatcharoen Hospital, Amnatcharoen, Thailand; b Division of Nephrology, Department of Medicine, King Chulalongkorn Memorial Hospital, Bangkok, Thailand; c Department of Microbiology, Faculty of Medicine, Chulalongkorn University, King Chulalongkorn Memorial Hospital, Thai Red Cross Society, Bangkok, Thailand; d Center of Excellence in Antimicrobial Resistance and Stewardship, Faculty of Medicine, Chulalongkorn University, Bangkok, Thailand; e Department of Infectious Diseases and Infection Control, Yamagata Prefectural Central Hospital, Yamagata, Japan; f Department of Infectious Diseases, Faculty of Medicine, Yamagata University, and Yamagata University Hospital, Yamagata, Japan; g Biofilms and Antimicrobial Resistance Consortium of ODA Receiving Countries, The University of Sheffield, Sheffield, UK; h Pathogen Hunter's Research Collaborative Team, Department of Infectious Diseases and Infection Control, Yamagata Prefectural Central Hospital, Yamagata, Japan; i Peritoneal Dialysis Excellent Center, King Chulalongkorn Memorial Hospital, Bangkok, Thailand; j Center of Excellence in Kidney Metabolic Disorders, Faculty of Medicine, Chulalongkorn University, Bangkok, Thailand

**Keywords:** *Eidernor doerrieniae*, *Eidernor*, Peritoneal dialysis, Fungal colonization, Catheter-related infections, Wet contamination

## Abstract

This report documents the first human case of *Eidernor doerrieniae* colonization in a peritoneal dialysis catheter, identified through DNA sequencing after a 52-year-old man observed brownish particles within his catheter. Despite the absence of peritonitis symptoms, prompt catheter removal and antifungal therapy successfully resolved the infection. Fungal cultures revealed cerebriform (brain-like) colonies, confirmed as *E. doerrieniae* using multi-targeted molecular diagnostics. A wet contamination event three weeks earlier was identified as the likely source. This case underscores the importance of recognizing intraluminal particles as an indicator of fungal colonization and highlights the critical role of timely intervention and advanced diagnostics in preventing fungal peritonitis.

## 1 Introduction

Subclinical intraluminal fungal colonization in peritoneal dialysis (PD) catheters is a significant yet underappreciated challenge in nephrology. While fungal colonization of implanted medical devices, such as intracardiac devices, has been increasingly documented [1], evidence for such colonization in PD catheters remains scarce, with only three cases reported to date, all presenting with visible intraluminal particles [[2], [3], [4]]. The subclinical nature of fungal colonization complicates diagnosis and may delay timely intervention [1], allowing fungi to proliferate and potentially progress to fungal peritonitis, a condition associated with high morbidity and mortality [5,6]. Recognizing visible particles in PD catheters, even in asymptomatic patients, may serve as a crucial clue for early diagnosis and management.

*Eidernor doerrieniae*, a basidiomycete fungus, has been isolated from environmental sources such as soil and insects [7] but has not previously been implicated in human infections. Here, we present the first documented case of *E. doerrieniae*-related PD infection, identified through DNA sequencing. This case underscores the importance of timely PD catheter removal upon identifying visible intraluminal fungal colonization, even in the absence of peritonitis symptoms. Furthermore, it highlights the critical role of molecular diagnostic techniques in identifying uncommon fungal pathogens associated with PD infections.

## 2 Case presentation

**Fig. 1:**
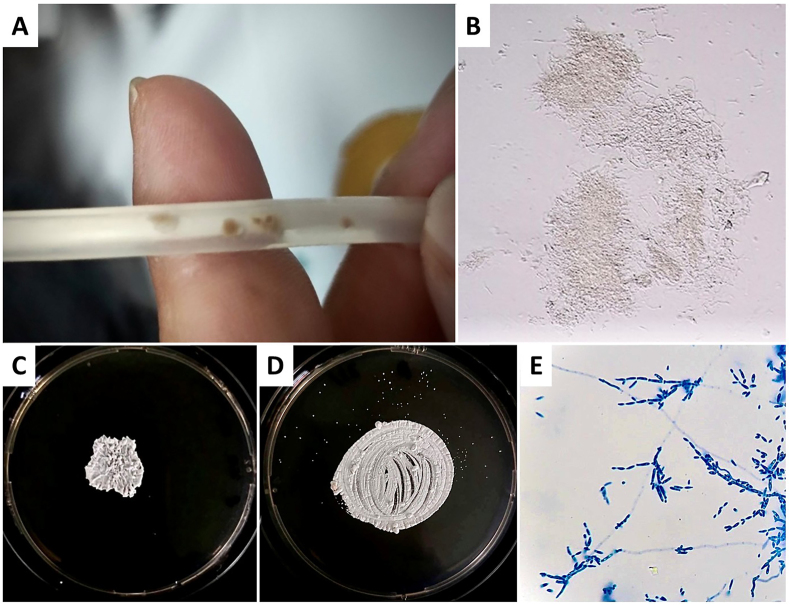
Fungal Colonization and Microbiological Features of *Eidernor doerrieniae* (A) Brownish particles visible inside the removed peritoneal dialysis (PD) catheter, suggestive of fungal colonization. (B) Potassium hydroxide (KOH) staining demonstrates hyphal structures under light microscopy (x400). (C) Early-stage colonies of *E. doerrieniae* on Sabouraud Dextrose Agar (SDA) at day 4, appearing white, smooth, and circular. (D) Mature colonies at day 7 exhibit a cerebriform (brain-like) surface with prominent furrows and a compact texture. (E) Lactophenol cotton blue staining shows pseudohyphal budding patterns with primary and secondary conidia under light microscopy (x400), supporting the identification of fungal growth.

A 52-year-old man with kidney failure on continuous ambulatory peritoneal dialysis (CAPD, 2L x 4 exchanges/day) for three years presented to the hospital after noticing brownish particles inside his PD catheter (May 2024, Day 0). He reported no abdominal pain or cloudy PD effluent (PDE). Physical examination revealed no fever, peritonitis, inflammation, or purulent discharge at the catheter exit-site, but brownish particles were visible within the catheter (Fig. 1A).

PDE analysis showed a leukocyte count of 16 cells/L, with 80 % mononuclear cells and 20 % neutrophils. Gram staining of the PDE was negative for organisms. Based on these findings, fungal colonization of the PD catheter was diagnosed. The PD catheter was promptly removed (Day 0), and the patient was transitioned to hemodialysis. Potassium hydroxide (KOH) staining of the particles inside the removed PD catheter revealed hyphal structures (Fig. 1B). Intravenous amphotericin B (40 mg/day) was administered for 14 days, followed by oral fluconazole (200 mg/day) for an additional 14 days.

Fungal culture of the PDE revealed colonies of *E. doerrieniae*, which initially appeared white, smooth, and circular with a slightly raised center during the early growth phase (2–4 days) on Sabouraud Dextrose Agar (SDA, Oxoid, Hampshire, UK) (Fig. 1C). By 7–10 days, the colonies matured into an off-white to beige coloration with a cerebriform (brain-like) surface, prominently furrowed and compact in texture (Fig. 1D and E). Initially reported as an "unidentified yeast-like fungus," definitive identification was achieved via DNA sequencing. Using large subunit (LSU = 28S) primers NL1/NL4, the sequence data confirmed the organism as *E. doerrieniae*, with 100 % query coverage and 99.8 % sequence identity (GenBank accession number NG_242143.1). Identification could not be established with internal transcribed spacer (ITS1/ITS4) or small subunit (SSU) primers (NS1/NS4). Additional genetic analysis confirmed the presence of the RNA polymerase II second largest subunit (RPB2) gene, whereas the tubulin gene was not expressed, underscoring the specificity of the molecular diagnostic approach.

Antifungal susceptibility testing of *E. doerrieniae* was performed using the Broth Dilution Antifungal Susceptibility Testing of Yeasts Method, following Clinical and Laboratory Standards Institute (CLSI) guidelines (Wayne, PA) [8]. The isolate was found to be susceptible to amphotericin B (MIC: 1 μg/mL) and isavuconazonium (1 μg/mL), while demonstrating resistance to voriconazole (1 μg/mL), posaconazole (1 μg/mL), fluconazole (>64 μg/mL), itraconazole (4 μg/mL), and caspofungin (>16 μg/mL).

Etiological investigation revealed that the patient, residing in northeast Thailand with a farming background, had been undergoing PD with the same catheter for three years, with the transfer set last exchanged six months prior. Importantly, the patient disclosed a wet contamination event occurring three weeks before presentation, which had not been reported to healthcare providers at the time. This incident was identified as the probable source of the fungal colonization. To mitigate future risks, comprehensive education on aseptic techniques and the critical importance of promptly reporting contamination events was provided to the patient.

## 3 Discussion

This case represents the first documented human infection caused by *E. doerrieniae*, identified through DNA sequencing after fungal colonization was observed as brownish particles within a PD catheter. Despite the absence of peritonitis symptoms, timely catheter removal guided by hyphal detection and antifungal therapy ensured infection resolution. A previously unreported wet contamination event was identified as the likely source, underscoring the importance of vigilant history-taking and patient education in preventing rare fungal infections in PD.

*E. doerrieniae*, a fungus classified under the phylum Basidiomycota, class Exobasidiomycetes, and order Exobasidiales, belongs to the genus *Eidernor* (NCBI:txid3068978) [7]. Previously identified strains include those isolated via Sanger sequencing from insect-derived specimens [[9], [10], [11]] and from soil samples [12]. Given the patient's occupation as a farmer, the pathogen was likely acquired through environmental exposure to soil or insects during PD bag exchanges. A suspected “wet contamination**”** event—defined as an open or unclamped system enabling microbial entry into the peritoneal cavity—occurred three weeks prior to the visual detection of fungal colonization. The identification of *E. doerrieniae* highlights the value of a multi-targeted molecular approach in detecting rare pathogens. While ITS1/ITS4, SSU, and tubulin primers failed to identify the organism, LSU (NL1/NL4) primers successfully confirmed its identity with high accuracy. Although RPB2 was positive, it did not contribute to species-level identification, underscoring the importance of employing multiple, targeted primers to enhance diagnostic precision in complex fungal cases.

The 2022 ISPD Peritonitis Guidelines [5], along with the 2009 IDSA guidelines for the diagnosis and management of intravascular catheter-related infections [13], primarily emphasize bacterial pathogens and symptomatic presentations. Reports of PD catheter fungal colonization without peritonitis are exceedingly rare, with only three documented cases, all presenting with visible intraluminal particles [[2], [3], [4]]. Two cases were managed successfully with catheter removal alone [3,4], while the third included adjunctive antifungal therapy [2]. All patients achieved favorable outcomes without relapses upon resuming PD [[2], [3], [4]]. However, one case initially treated with a transfer set exchange relapsed within two weeks, underscoring the limitations of conservative approaches [3].

Additionally, two reports documented asymptomatic intraluminal fungal colonization preceding peritonitis by 4 and 7 days, caused by *Aureobasidium pullulans* [14] and *Sporothrix schencki* [15], respectively. These cases highlight the potential for fungal colonization to progress to peritonitis if not promptly addressed. Although evidence is limited, timely PD catheter removal and antifungal therapy, even in asymptomatic cases, appear crucial to prevent severe outcomes. Delays in catheter removal or attempts at salvage have consistently resulted in poor outcomes, reinforcing the need for decisive intervention [6,16,17].

In line with the 2022 ISPD Peritonitis Guidelines [5], immediate PD catheter removal followed by at least 14 days of antifungal therapy is recommended for fungal infections. While typically applied to symptomatic fungal peritonitis, this approach should also extend to asymptomatic colonization to prevent progression and adverse outcomes. Visible intraluminal particles in a PD catheter, even in the absence of peritonitis symptoms, serve as a critical clue to fungal colonization. Early and decisive management remains essential to mitigating risks in these high-risk scenarios. Recognizing the subclinical presence of fungal pathogens underscores the importance of reliable detection protocols and effective surveillance mechanisms to improve patient outcomes.

This case underscores the importance of recognizing visible intraluminal particles as a critical indicator of fungal colonization, even in the absence of peritonitis symptoms. Immediate catheter removal and timely antifungal therapy remain essential to preventing progression to fungal peritonitis. Proactive surveillance following wet contamination events could facilitate early detection of subclinical fungal colonization and enable prompt intervention. The identification of *E. doerrieniae* in this case, the first documented human infection, highlights the value of a multi-targeted molecular approach, including LSU primers, in diagnosing rare fungal pathogens. This emphasizes the need for heightened vigilance and comprehensive diagnostic strategies, particularly in PD patients with environmental exposure risks.

## CRediT authorship contribution statement

**Phichit Songviriyavithaya:** Writing – review & editing, Writing – original draft, Conceptualization. **Aschariya Wipattanakitcharoen:** Data curation. **Niparat Pikul:** Data curation. **Dhammika Leshan Wannigama:** Writing – review & editing. **Talerngsak Kanjanabuch:** Writing – review & editing, Supervision, Funding acquisition, Formal analysis, Conceptualization.

## Declaration of competing interest

This research was generously supported by several esteemed institutions: the 10.13039/501100010724Health Systems Research Institute (10.13039/501100010724HSRI) [Grant No. 67-130], Thailand; the 10.13039/501100017170Thailand Science Research and Innovation Fund, 10.13039/501100002873Chulalongkorn University, Thailand [Grant Nos. HEAF67300066 and HEA_FF_68_018_3000_002]; and the Ratchadapiseksompotch Fund, 10.13039/501100002873Chulalongkorn University, Thailand [Grant Nos. HEA663000115 and HEA663000116]. Further support was provided by the Ratchadapiseksompotch Fund, Graduate Affairs, 10.13039/501100004776Faculty of Medicine, Chulalongkorn University, Thailand [Grant No. GA66/047]; the Kidney Foundation of Thailand [Grant No. 1205/2564]; and the 10.13039/100020648Royal College of Physicians of Thailand [Grant No. 02/66]. TK has received consultancy fees from VISTERRA and AstraZeneca as a country investigator and is a recipient of funding from the National Research Council of Thailand. Additionally, TK has received speaking honoraria from AstraZeneca, Alexion Pharmaceuticals Inc., Fresenius Medical Care, and Baxter Healthcare. The remaining author has declared no commercial or financial relationships that could be perceived as potential conflicts of interest related to this article.
